# Hallucinations: A Systematic Review of Points of Similarity and Difference Across Diagnostic Classes

**DOI:** 10.1093/schbul/sbw132

**Published:** 2016-11-21

**Authors:** Flavie Waters, Charles Fernyhough

**Affiliations:** ^1^School of Psychiatry and Clinical Neurosciences, The University of Western Australia, Perth, Western Australia, Australia;; ^2^Clinical Research Centre, Graylands Hospital, North Metro Health Service–Mental Health, Perth, Western Australia, Australia;; ^3^Hearing the Voice, c/o School of Education, Durham University, Durham, UK;; ^4^Department of Psychology, Durham University, Durham,UK

**Keywords:** schizophrenia, bipolar disorder, psychosis, nonclinical

## Abstract

Hallucinations constitute one of the 5 symptom domains of psychotic disorders in DSM-5, suggesting diagnostic significance for that group of disorders. Although specific featural properties of hallucinations (negative voices, talking in the third person, and location in external space) are no longer highlighted in DSM, there is likely a residual assumption that hallucinations in schizophrenia can be identified based on these candidate features. We investigated whether certain featural properties of hallucinations are specifically indicative of schizophrenia by conducting a systematic review of studies showing direct comparisons of the featural and clinical characteristics of (auditory and visual) hallucinations among 2 or more population groups (one of which included schizophrenia). A total of 43 articles were reviewed, which included hallucinations in 4 major groups (nonclinical groups, drug- and alcohol-related conditions, medical and neurological conditions, and psychiatric disorders). The results showed that no single hallucination feature or characteristic uniquely indicated a diagnosis of schizophrenia, with the sole exception of an age of onset in late adolescence. Among the 21 features of hallucinations in schizophrenia considered here, 95% were shared with other psychiatric disorders, 85% with medical/neurological conditions, 66% with drugs and alcohol conditions, and 52% with the nonclinical groups. Additional differences rendered the nonclinical groups somewhat distinctive from clinical disorders. Overall, when considering hallucinations, it is inadvisable to give weight to the presence of any featural properties alone in making a schizophrenia diagnosis. It is more important to focus instead on the co-occurrence of other symptoms and the value of hallucinations as an indicator of vulnerability.

## Introduction

The personal, cultural, and clinical significances of hallucinations have changed in the 200 years since they were defined by Esquirol as “the intimate conviction of actually perceiving a sensation for which there is no external object.”^1,2^ There is a growing recognition that hallucinatory experiences attend a wide variety of psychiatric diagnoses and can be part of everyday experience for people who do not meet criteria for mental illness. For the experiencer, hallucinations can have important personal meanings, and for clinicians, they are also significant in varied ways, including as diagnostic symptom and as factors which may impact on functioning and prognosis, and therefore potentially the necessity of treatment.

Since the earliest clinical texts, auditory hallucinations have been closely linked to schizophrenia.^3,4^ Hallucinations constitute one of the 5 domains of abnormality of schizophrenia spectrum and other psychotic disorders in DSM-5. Although not diagnostic of schizophrenia when occurring in isolation, the presence of persistent auditory hallucinations is sufficient for a diagnosis of Other Specified Schizophrenia Spectrum and Other Psychotic Disorder, if it is combined with clinically significant distress or functional impairment (ref.^5^, p. 122).

The emphasis on the clinical significance of hallucinations in DSM-5^5^ requires closer examination given our growing understanding of hallucinations that feature outside of psychosis. It is increasingly recognized that hallucinations occur with significant frequency in other psychiatric (eg, post-traumatic stress disorder [PTSD], personality disorders) and medical conditions (neurodegenerative conditions and eye disease) and that they are especially predictive of multimorbid psychopathology.^6,7^ A predominant view, however, is that some features of hallucinations may distinguish schizophrenia and related disorders from other conditions. Candidate distinguishing features that have become important diagnostically include negative and derogatory auditory hallucinations; command hallucinations; voices heard conversing about the individual in the third person; and location in external space.^4,5,8^

Given the status of hallucinations as diagnostic markers in DSM-5, there is a pressing need to establish their specificity to psychotic disorders, particularly through comparison of their featural properties across different population groups. This exercise is also essential if we are to understand how hallucinations function in cognitive and neuroscientific models of psychiatric disorders such as schizophrenia.

The present article attempts to meet this challenge through a systematic review of empirical studies that have made direct comparisons of hallucinations involving 2 or more population groups (one of which included schizophrenia). Our focus is on visual and auditory hallucinations, because these experiences figure more prominently in schizophrenia research than other modalities of hallucination. We place particular emphasis on whether hallucinations share points of commonality or divergence between different groups. Because diagnostic criteria inevitably focus on self-report rather than objective markers, the bulk of studies have reported on the phenomenological features of hallucinations. Too few studies have directly compared cognitive or neuroimaging variables between 2 and more groups for meaningful analyses, although helpful syntheses of the literature exist.^9–12^ In this article, we present a direct contrast of hallucination features across clinical groups with a view to identifying features specific to schizophrenia. First, we briefly consider the different conditions and groups in which hallucinations have been reported.

## Disorders and conditions in which hallucinations present

There are many disorders and conditions (including nonpathological cases) in which hallucinations have been reported (supplementary material table 1, adapted^13–15^).

Psychotic experiences occur in around 4%–7% of the general population,^16,17^ proving transitory in around 80% of cases.^18^ In this group, incidence of hallucinations varies with developmental stage, appearing with relatively high prevalence in children (8%^19^) and older adults (community 1%–5%; residential care 63%^20^), and some adults on a continuum of hallucination proneness. Hallucinations also occur transiently in situations causing extreme physiological or psychological stress (fatigue, sensory deprivation, bereavement, etc.).^21,22^

Hallucinations co-occur with toxin-, alcohol-, and drug-related conditions that affect the central nervous system and involve the cortical sensory areas. These include intoxication with stimulants, hallucinogenic drugs, and cannabis, and withdrawal from substances including alcohol.^23^ Medication-induced hallucinations also result from drug abuse or occur as side effects of attention-deficit/hyperactivity disorder drugs,^24^ antimalarial medication,^25^ and antiparkinsonian drugs. In these cases, hallucinations usually disappear after withdrawal of the substance.

Hallucinations are associated with a range of medical conditions. Conditions causing interference with, or damage to, the peripheral sensory pathways can produce hallucinations. For example, acquired deafness is a common cause for (auditory) hallucinations, as is eye disease or lesions of afferent visual pathways for visual hallucinations.^26,27^ Endocrine-related metabolic disorders including disorders of thyroid function^28^ and Hashimoto disease^29^ can produce hallucinations, as can deficiencies in D and B12 vitamins.^30,31^ Other medical conditions associated with hallucinations include chromosomal disorders such as Prader–Willi syndrome,^32^ autoimmune disorders,^33^ and acquired immunodeficiency disorders such as HIV/AIDS,^34^ and sleep disorders such as narcolepsy.^35^ Neurological events such as tumors,^36,37^ traumatic brain injuries,^38^ epilepsy,^39^ and cardiovascular events may also cause hallucinations where activity involves the brainstem regions and areas involving the temporal, occipital, or temporo-parietal pathways. Hallucinations are also fairly common in neurodegenerative conditions, particularly in Parkinson’s disease^40^ and dementia with Lewy bodies.^41^

Finally, hallucinations are reported in a range of major psychiatric conditions. In addition to psychotic disorders (schizophrenia, schizotypal personality traits, schizoaffective disorders, other specified spectrum disorders, etc.), hallucinations and other psychotic experiences occur in affective disorders such as bipolar (mixed, manic, depressed) and unipolar depression^42^; personality disorders; PTSD; and anorexia and bulimia nervosa.^43^

In summary, because hallucinations occur in a number of different clinical groups, they can be considered nonspecific for psychotic disorders. A categorical approach reliant solely on the presence of hallucinations is therefore not helpful as a diagnostic aid.

## How do hallucinations present across diagnostic classes?

In this section, we consider data as they inform on points of commonality and divergence across diagnostic classes of psychiatric illness, with a particular focus on comparisons with schizophrenia spectrum disorders. A predominant aim is to determine which, if any, features of hallucinations are pathognomonic: that is, specific to schizophrenia (as the “prototypical” psychotic disorder) and not reported in any other population group.

### Methods

**Fig. 1.**
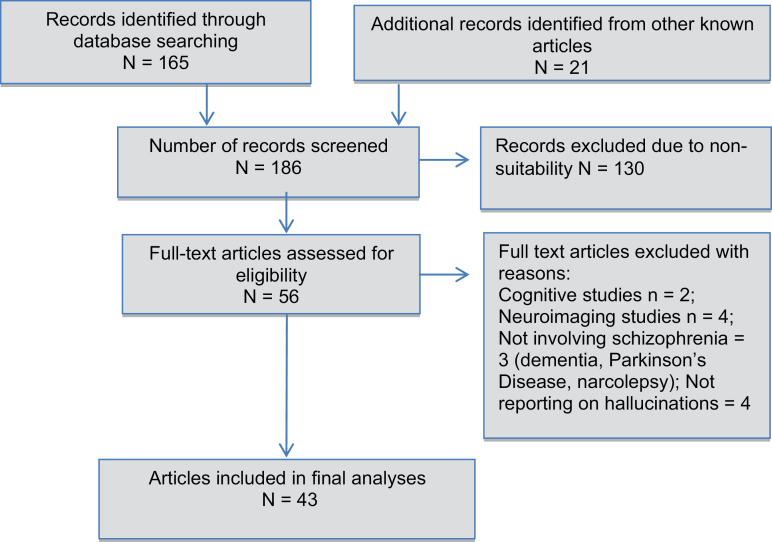
PRISMA diagram.

To answer these questions, a search was carried out in Medline for English-language articles and book chapters containing the following terms (Hallucinat* OR Psychot* Symptom*) AND (Compar* OR Differen* OR Similarit* OR Contrast*), without date restrictions. Inclusion criteria included empirical articles involving direct contrasts between 2 and more population groups with hallucinations, where one of these groups included schizophrenia-spectrum disorders. Exclusion criteria were as follows: single case studies and reviews, studies reporting on “hallucination proneness” as a continuous variable, and publication in languages other than English. The search was conducted on June 20, 2016, and revealed 165 articles, to which 21 additional articles were added from known articles and cross-referencing. Title and abstracts, and subsequently the entire articles, were scanned to ascertain whether they fulfilled the criteria (see figure 1 for PRISMA diagram). A total of 56 articles met the criteria.

The great majority of articles (90.1%) demonstrated a focus on featural or clinical characteristics of hallucinations (supplementary material table 2). The remaining articles reporting solely on imaging data (*n* = 4) or cognitive processes (*n* = 2) were excluded. Seven further articles were excluded for not involving a schizophrenia group or not reporting on hallucinations specifically.

In total, 43 articles were reviewed (see table 1). Articles involving direct comparisons with schizophrenia included the following 4 major groups: (1) nonclinical groups (general population,^44–48^ religious experiences^49,50^; total 7 studies); (2) drug- or alcohol-related (substances) conditions^51–59^ (9 studies); (3) medical and neurological conditions (tinnitus,^60^ epilepsy,^61,62^ narcolepsy,^35^ other medical and neurological causes^53,63–66^; total 9 studies); and (4) psychiatric disorders (affective disorders,^8,42,53,64,66–70^ borderline personality disorder,^71–74^ PTSD,^75,76^ dissociative identity disorder,^44,77^ anorexia^78^; total 18 studies). There were a further 2 articles on attenuated psychosis (ultra high risk [UHR]),^79,80^ which were classed in the psychiatric group. Noticeably absent were direct comparisons with Parkinson’s disease and other neurodegenerative conditions. Given this important gap, we chose to include one recent review comparing visual hallucinations in schizophrenia to those in Parkinson’s disease, Lewy body dementia, and eye disease^81^ (classed in the medical and neurological category).

**Table 1.** Phenomenological Features of Hallucinations Across 4 Population Groups (%) and Comparisons With Schizophrenia (Mean %^82^)

Characteristic Features of Hallucinations	Schizophrenia	Nonclinical	Substances	Medical, Neurologic	Other Psychiatric	Groups Showing the Same Features as Schizophrenia
**Form**						
Presence of auditory verbal hallucinations (“voices”)	75%	±58%	±50%	±32%	46%–57%	Nonclinical^44,45,48^ (including evangelical born-again Christians^50^); alcohol dependence disorder,^51,56^ cocaine withdrawal^55^; tinnitus,^60^ epilepsy,^62,83^ medical or neurological condition,^63^ narcolepsy^35^; Parkinson’s disease^81^; bipolar disorder,^42,69,70^ borderline personality disorder,^73,74^ dissociative identity disorder,^77^ PTSD^75^
Predominance of auditory (A) over visual (V) hallucinations	75% A, 30% V	**Nil**	**Nil**	±32% A, ±10% V	±28% A, ±15% V	Temporal lobe epilepsy,^62,83^ medical or neurological condition^63,84^; bipolar disorder^42,^^67^
Hallucinations in 3 or more sensory modalities	60%	**Nil**	±16%	±20%	±76%	Cocaine abuse,^55^ LSD intoxication,^54^ alcohol dependence disorder^53^; bipolar disorder,^66,70^ dissociative identity disorder,^77^ narcolepsy^35^
Duration: up to several hours at a time	66%	±47%	?%	±70%	77%–93%	Nonclinical^44^ (including evangelical born-again Christians^50^); alcohol dependence disorder,^53^ cocaine abuse^55^; tinnitus,^60^ temporal lobe epilepsy^62^; dissociative identity disorder,^44^ borderline personality disorder^73^
Perceived to be vivid and real	80%	?%	±26%	±48%	54%–100%	Nonclinical^28,44,48^; alcohol dependence disorder,^51^ cocaine abuse^55,56,59^; LSD intoxication^58^; tinnitus,^60^ temporal lobe epilepsy,^62,83^ medical or neurological condition^63^; PTSD,^76^ bipolar disorder,^64,66,69^ dissociative identity disorder,^44,77^ borderline personality disorder^72–74^
Originating in external space	50%	±57%	±70%	±37%	60%–83%	Nonclinical^28,44^; alcohol dependence disorder^51^; tinnitus,^60^ medical or neurological condition^63^; dissociative identity disorder,^77^ PTSD,^76,77^ bipolar disorder,^8,64,66,69^ borderline personality disorder^72–74^
Third-person hallucinations, running commentary, voices commenting	65%	20%–41%	20%–60%	10%–41%	40%–80%	Nonclinical^28,44^; cannabis abuse^57^; alcohol dependence disorder^53,56^; medical or neurological condition,^63,84^ temporal lobe epilepsy^62,83^; affective disorders,^69^ dissociative identity disorder,^44^ PTSD,^76^ borderline personality disorder,^74^ narcolepsy^35^
Hallucinations shared with other people (eg, voices “heard” by others)	50%	**Nil**	**Nil**	±62%	±27%	Epilepsy,^84^ neurological condition^66^; bipolar disorder^66,69^
**Contents**						
Negative and hostile content	60%	43%–53%	?%	±33%	58%–93%	Nonclinical^44,48^ (including evangelical born-again Christians^50^); cocaine withdrawal,^55^ alcohol dependence disorder^56^; temporal lobe epilepsy^62^; Parkinson’s disease^81^; medical and neurological condition,^63^ affective disorders mixed^69^; PTSD,^75,76^ borderline personality disorder^72,73^; dissociative identity disorder^44,77^
Personifications and attributions to spiritual or magical identities	61%	50%–100%	20%–60%	±50%	±97%	Nonclinical^28,46^ (including evangelical born-again Christians^50^); cocaine withdrawal^55^; alcohol dependence disorder^56^; medical or neurological condition^63,64^; temporal lobe epilepsy^62^; PTSD^76^
Assigned significance (eg, hallucinations have meaning)	72%	?%	±42%	±76%	?%	Nonclinical (including evangelical born-again Christians^50^ and other religious voice-hearers^49^); cocaine withdrawal,^55^ LSD intoxication^54^; temporal lobe epilepsy^62^; bipolar disorder,^64^ borderline personality disorder^71^
Commands to commit aggressive or injurious acts	84%	**Nil**	±4%	?%	62%–82%	Amphetamine withdrawal and dependence,^52,55^ alcohol substance disorder^56^; temporal lobe epilepsy^62^; bipolar disorder,^69^ PTSD,^76^ dissociative identity disorder^77^
**Interference and lack of control**						
Interference with daytime functions	47%	**Nil**	±30%	?%	±84%	Cocaine withdrawal^55^; tinnitus^60^; temporal lobe epilepsy^62^; PTSD,^75,76^ borderline personality disorder,^72,73^ bipolar disorder,^64^ narcolepsy^35^
Lack of perceived control	78%	**Nil**	?%	±53%	±78%	Alcohol substance disorder^51^; tinnitus,^60^ temporal lobe epilepsy,^62,83^ medical or neurological condition^64,66^; borderline personality disorder,^72,73^ bipolar disorder^66,69^
**Other clinical and epidemiological features**						
Recurrent course of hallucinations (>1 y, multiple episodes)	60%	±89%	**Nil**	?%	±35%	Nonclinical^47,48^; epilepsy^62,83^; PTSD,^76^ bipolar disorder^68,85^
Hearing or vision loss	?%	**Nil**	**Nil**	?%	?%	Tinnitus,^60^ medical or neurological condition,^65,66^ neurodegenerative disease^81^; UHR psychosis^79^
Trauma and neglect	50%	±50%	**Nil**	**Nil**	±50%	Nonclinical population^44,47^; dissociative identity disorder^44,77^; borderline personality disorder^72,73^
External triggers	50%	**Nil**	**Nil**	**Nil**	±80%	Affective disorders,^69^ dissociative identity disorder^44,77^
Internal triggers	60%	?%	?%	?%	±60%	Nonclinical population^44^; tinnitus^60^; all substances are internal triggers; PTSD^76^
Family history of psychiatric illness	30%	**Nil**	±23%	±30%	±40%	Epilepsy^83^; cannabis abuse^57^; anorexia nervosa^78^
Age of onset in late teen to early 20s	18–24	**Nil**	**Nil**	**Nil**	**Nil**	
**Feature commonality with schizophrenia (number and %**)	n/a	11/21 = 52%	14/21 = 66%	18/21 = 85%	20/21 = 95%	

*Note:* n/a, not applicable; LSD, lysergic acid diethylamide; UHR, ultra high risk. “Nil” findings are shaded in bold. “?%” indicates that percentages were not reported in the articles reviewed.

### Results

Findings are organized around the 21 most commonly described features of hallucinations in schizophrenia, sorted according to modality, form, content, interference, and lack of control,^86^ and other clinical and epidemiological features. Percentages (when available) are shown in table 1. An entry of “nil” indicates that this feature has not been reported in this diagnostic group.

Overall, psychiatric disorders shared the most features with schizophrenia (20/21, 95%); followed by medical/neurological disorders, with 18/21 features of commonality (85%); and by substance-related conditions (11/21, 66%). Nonclinical groups shared approximately half of all features (11/21, 52%).

Auditory (verbal and other sounds) hallucinations occurred in all 4 major groups, with the highest prevalence (outside schizophrenia) reported in evangelical born-again Christians (58%)^50^ and bipolar disorder (57%).^42^ The predominance of auditory hallucinations over other modalities (eg, visual hallucinations) is commonly viewed as indicative of schizophrenia,^5^ although this profile is also reported in medical conditions including temporal lobe epilepsy,^83^ as well as in bipolar disorder.^42^

Multiple modalities of hallucinations (>3, although not simultaneously) were originally thought to be distinctive for schizophrenia,^64,66^ but this pattern also occurs in the context of drugs and alcohol,^55^ and narcolepsy.^35^ Psychiatric conditions such as bipolar disorder and dissociative identity disorder^77^ report several modalities of hallucinations (typically 2 or 3), but generally fewer than schizophrenia (where individuals may report 4 or 5 distinct modalities).

Specific formal parameters of hallucination (persistent voices, vividness and realism, and origin in external space) are also displayed in the 4 major groups. For example, continuous voices over several hours among those who have hallucinations have been reported in nonclinical individuals at rates of 47%,^44^ tinnitus (70%),^60^ dissociative identity disorder (93%^44^), and affective disorders (77%^69^). Similarly, (visual) hallucinations in Parkinson’s disease may last for hours at a time.^81^ The experience is highly vivid and perceived to be real in 26% of alcohol withdrawal cases,^56^ bipolar disorder (100%^69^), and PTSD (54%^76^). Voices originating in external space are not pathognomonic of schizophrenia, occurring at rates of 57% in nonclinical groups,^44^ 37% in tinnitus,^60^ 60% in PTSD,^76^ 83% in bipolar disorder,^64,66,69^ and 67% in dissociative identity disorder.^44^

Third-person hallucinations and voices commenting or conversing with each other do not distinguish between groups. Voices commenting are reported in nonclinical groups (41% of those who hear voices)^44^ and in patients with narcolepsy (17%),^35^ dissociative identity disorder (80%),^44^ and affective disorders (54%).^69^ Similarly third-person hallucinations are reported at rates of 20% in nonclinical groups,^44,45^ 10% in narcolepsy,^35^ 25%–60% in alcohol withdrawal,^53,56^ and 40% in affective disorders.^69^ Approximately one-third of individuals with schizophrenia expect their voices to be heard by others,^87^ a feature additionally observed in bipolar disorder and organic psychosis.^66,84^

With regard to contents, negative, accusatory, or derogatory voices occur in all groups, eg, 93% of individuals with dissociative identity disorder with hallucinations, 58% of affective disorders,^69^ and 62% of PTSD.^76^ Negative contents are also common in both auditory and visual hallucinations in alcohol withdrawal^56,88^ and in neurodegenerative conditions.^81^ Negative voices occur less frequently in nonclinical groups, although they are still reported in approximately half^44,48^ of these individuals.

Similarly, personification and attribution of hallucinations did not distinguish between groups. Nonclinical groups often attribute their hallucinations to a relative or acquaintance,^45^ to deceased persons, and to religious figures,^46^ whereas personification to supernatural entities is more common in religious experiences,^50^ substance-related conditions,^55^ and psychiatric disorders.^76^ In Parkinson’s disease and dementia, hallucinations are attributed to people who may be familiar or not, alive or deceased, or to guardian angels.^81^

Hallucinations assigned special significance are common in the context of drugs,^54^ temporal lobe epilepsy,^62^ bipolar disorder,^64^ and borderline personality disorder.^71^ Such special significances are also reported in religious experiences,^49,50^ although the interpretation is more positive than for those in schizophrenia.

Voices giving commands to commit aggressive or injurious actions are observed in most groups. For example, in a PTSD sample, 62% of those hearing voices were commanded to self-harm, and 46% had acted on command hallucinations.^75,76^ Command hallucinations are also reported in 82.3% of individuals with bipolar disorder.^69^ No data are reported on this feature in nonclinical individuals.

Interference with daily life, and lack of perceived control, is a prominent issue in all but nonclinical groups. For example, daytime interference is reported in 84% of individuals with dissociative identity disorder.^44^ Similarly, lack of control over the onset and content of hallucinations is common with substance-related, medical, and neurological causes,^60^ as well as borderline personality disorder^72^ and bipolar disorder.^44^ By contrast, nonclinical groups report significantly less distress or daytime interference than individuals with schizophrenia,^44^ and greater levels of control.^45^

A persistent course of hallucinations (lasting for more than 1 year or multiple episodes) has been demonstrated in nonclinical groups,^47^ epilepsy,^83^ bipolar disorder,^85^ and PTSD in which hallucinations can persist for longer than 1 year in the majority of cases.^76^ In Leudar and coworkers’ sample of 13 nonclinical individuals,^48^ recurrent voice hallucinations were reported in 89% and had been ongoing on average for longer than 4 years.

Finally, shared epidemiological and clinical features of hallucinations include the following: (1) hearing or vision loss as a vulnerability factor for hallucinations, which is additionally reported in UHR groups,^79^ neurological conditions including tinnitus,^60^ and neurodegenerative conditions such as Parkinson’s disease^81^; (2) negative life events, such as emotional neglect and trauma, reported in nonclinical groups (50%)^44^ and psychiatric conditions including dissociative identity disorder (50%)^44,77^ and borderline personality disorder^72,73^; (3) triggers that include exogenous (external) situations in dissociative identity disorder (at rates of 80%^44^) and affective disorders^69^ but not nonclinical groups^44^, and endogenous (internal) triggers such as sadness, stress, and tiredness reported in the nonclinical group,^44^ PTSD (60%),^76^ and tinnitus^60^; and (4) family history of psychiatric illness, which is displayed across all major groups except nonclinical groups.

One feature of hallucinations appears to distinguish schizophrenia from other groups. Age of onset of hallucinations in schizophrenia is typically from the late teens to early 20s. Direct comparison demonstrates that an earlier age of onset (<12 years) is more frequent in dissociative identity disorder (33%) and nonclinical groups (40%) than in schizophrenia (11%).^44^ Daalman et al^45^ showed a mean age of onset of 12.4 years for nonclinical individuals compared to 21.4 years in schizophrenia. Hallucinations in affective disorders, neurological disorders, and alcohol-related conditions, by contrast, appear predominantly in middle or older age.^56,62^

## Discussion of findings

Our systematic review demonstrates that almost all phenomenological, clinical, and epidemiological features of hallucinations in schizophrenia are also represented in other population groups, with the sole exception of age of onset.

### Points of similarity and difference among diagnostic classes

Our findings did not support the notion that some features of hallucinations are uniquely diagnostic of schizophrenia.^4,8,89^ Auditory “verbal” hallucinations, which are vivid, occur frequently, and are experienced as coming from outside of the head, are present in all 4 major groups considered here (nonclinical, substances, medical or neurological, and psychiatric). Examples include hallucinations linked to religious experiences,^49,50^ dissociative identity disorder,^44,77^ and temporal lobe epilepsy.^62,90^ Of note, voices originating in external space occurred in half of nonclinical voice hearers,^44^ one-third of individuals with tinnitus,^60^ and 60%–83% of individuals with PTSD,^76,77^ bipolar disorder,^69^ and dissociative identity disorder.^44^ The highlighting of Schneider’s first-rank symptoms (including third-person hallucinations and voices conversing) as indicative of schizophrenia^4^ was not supported, with these features occurring in nonclinical samples at rates of 20%–40%,^44,45^ narcolepsy (10%–17%),^35^ alcohol withdrawal (26%–60%),^53,56^ affective disorders (20%–55%),^69^ and dissociative identity disorder (80%).^44^

Similarly, the widely held notion that negative voices are indicative of psychosis^91^ was not confirmed. Negative and derogatory (visual and/or auditory) hallucinations occurred in all (clinical and nonclinical) groups. Similarly, the way in which hallucinations are personified and assigned special significance (ie, seen as personally meaningful) is also a feature of other psychiatric conditions (bipolar disorder, borderline personality disorder, PTSD)^64,71,76^ and temporal lobe epilepsy.^62^ In nonclinical groups (such as evangelical born-again Christians), it is common for voices to be interpreted as having religious meaning.^49,50^

One feature of interest is hallucination frequency, which is often difficult to assess empirically (e.g., ref.^50^) and is confounded both with symptom incidence and hallucination duration (see table 1). Although some studies show hallucinations to be more frequent in schizophrenia in relation to a comparison group (ref.^10,80^), others show hallucinations in comparison groups to have an objectively high rate of occurrence (e.g., refs^66,69,76^). In the absence of further data, frequent hallucinations can thus not be considered to be a diagnostic feature of schizophrenia. Altogether, the featural properties of hallucinations carry minimal information about the condition in which it occurs.

Only one feature, age of onset, was identified as of potential interest for distinguishing schizophrenia from other groups. We note, however, that this relates to a continuous variable rather than a clear presence or absence of any hallucination feature. Its diagnostic value is further limited given the small number of studies that have considered it transdiagnostically, the potential confound of prodromal symptoms predating the onset of frank psychosis, potential unreliability of reports due to difficulties in remembering a first experience that might have occurred many years ago, and the relatively high variance in age of onset even in schizophrenia.

With regard to points of rarity among other groups, one feature deserving of more research concerns external triggers for hallucination. External factors including a lack or an excess of external stimulation (eg, social contact), are a trigger in 80% of schizophrenia cases,^82^ and similar rates have been reported in dissociative identity disorder and affective disorders.^44,69^ By contrast, there is an absence of information regarding external triggers in nonclinical groups on the basis of the one direct comparison study reviewed here.^44^ We note however that one small qualitative study investigating “out-of-the-ordinary” experiences including hallucinations^92^ showed triggering by social isolation.

### Points of rarity for major diagnostic groups

We also examined points of rarity for each major group. A historical focus on the symptoms of psychosis has reinforced a “schizophrenia-centric” approach to hallucination descriptions, yet our analysis allows an examination of each disorder group individually. Specifically, table 1 enables an inspection of how hallucination features are shared among diagnostic groups, indicating possible points of rarity.

With regard to hallucinations in psychiatric disorders (particularly affective disorders, borderline personality disorder, dissociative identity disorder, and PTSD), table 1 suggests that no hallucination feature differentiated them from schizophrenia except age of onset (95% feature commonality). This supports findings of studies reporting on the overlap in genetic, neurobiological, and clinical features between schizophrenia and these psychiatric disorders.^93,94^

Hallucinations caused by the abuse or withdrawal of substances (toxins, drugs, alcohol) differ from schizophrenia in the following features: visual hallucinations are more common than auditory hallucinations; the course is not recurrent but proximally related to substance ingestion; and hearing or vision loss is not a cause.

A discussion on hallucinations in medical and neurological conditions is complicated by the heterogeneity of this group (incorporating tinnitus, epilepsy, narcolepsy, neurodegenerative conditions, and other medical and neurological conditions). Interestingly, conditions such as epilepsy and tinnitus share up to 85% (18/21) of features with schizophrenia and appear almost indistinguishable in form and content,^60,62,84,90^ except for clinical and epidemiological correlates such as trauma and external triggers. The absence of information on triggers may merely reflect the lack of direct comparison studies reporting on this issue. For example, photic stimulation, TV, and video games are triggers for complex partial seizures.^95^ Overall, too few direct comparisons exist for confident conclusions regarding unique hallucination features in medical and neurological conditions.

Reflecting its relative newness as a field of research attention, only 7 studies involving nonclinical individuals met inclusion criteria. Hallucinations in this group shared 11 out of 21 features with schizophrenia (52%), including vivid and frequent voices, third-person hallucinations, personification, a recurrent course of hallucinations, and an increased risk for adverse negative events. A potential point of rarity distinguishing this nonclinical group is that such experiences receive a more positive interpretation compared to psychiatric disorders. Although contents may be negative^96^ in up to 50% of cases,^44^ voices are more often positive (93% of cases compared with 83% in schizophrenia and 67% in dissociation identity disorder) and are largely controllable. The affective reaction to voices is also more positive in the nonclinical group. In the sample of evangelical born-again Christians,^50^ voices are actively encouraged and given positive religious meanings. Such positive interpretations are presumably consistent with reported reductions in daytime interference, agitation, and fear, and with the fact that such individuals do not seek care. Several studies have now described the profile of nonclinical voice hearers as varying on a continuum with voices in schizophrenia.^10,47^ Additional differences identified here include that (1) other modalities of hallucination (eg, visual or body related) may be as common as voices (or even more so); (2) such individuals experience a lesser total number of different modalities of hallucinations (<3); and (3) hearing loss or visual deficits do not appear as a frequent cause.

Finally, table 1 was examined to establish whether any hallucination features are shared among all but one diagnostic groups, suggesting a point of rarity for that exception group. This criterion was met only for the nonclinical group. Four out of 21 (21%) hallucination features were shared among all other groups except this one (hallucinations in ≥3 sensory modalities, commands to commit aggressive or injurious acts, interference with daytime functions, and lack of perceived control).

### Interaction between features

The above analysis suggests that featural properties of hallucinations alone are unlikely to be able to support a diagnosis of schizophrenia. More important than the presence or absence of any specific hallucination features, however, is arguably the question of how those features interact with other symptoms. Two issues need to be distinguished here: the question of whether the specific profile of symptoms and symptom features creates distinctive signatures for different diagnoses, and the question of how symptoms interact as they co-occur. In relation to the first question, table 1 indicates how a transdiagnostic comparison of hallucination features may indicate distinctive profiles for particular disorders. Clearly, in distinguishing schizophrenia from (say) toxin-related disorders, other clinical information (such as patient history of toxin use) will be highly relevant. Further research in transdiagnostic comparison of symptom features (beyond their mere absence or presence) may therefore be of value in improving differential diagnosis.

With regard to the second question, there has recently been considerable interest in the view that psychotic symptoms occur transdiagnostically and that features of psychosis may occur on a continuum as an extended phenotype in the general population.^7^ With the application of methodologies such as graph theory to psychosis research, new methodologies are becoming available to better understand psychosis as it operates within a network of interacting symptoms.^97^ Most importantly, such methods demonstrate how, through investigation of interactions between variables, it is possible to understand how hallucinations may function or impact differently in different diagnostic groups according to what roles they play in the network.

## Clinical applications

This review yields an important conclusion with regard to the diagnostic value of hallucinations. It concludes that it is inadvisable to give weight to the presence of any featural properties of hallucination alone when making a diagnosis. For example, negative and hostile voices talking in the third person and causing distress may equally be present in a patient with schizophrenia, epilepsy, brain tumor, or PTSD. However, this conclusion does not entail that hallucinations are clinically uninformative. First, clinical observation suggests that some conditions have their own distinctive hallucination signature providing clues regarding their underlying causes (eg, “cocaine bugs”^55^). Second, the greater clinical significance of hallucinations may lie in their value in designating the severity of psychopathology. The presence of hallucinations indicates increased risk of poor outcomes transdiagnostically, including multimorbid nonpsychotic disorders, suicidality, neurocognitive deficits, and low functioning.^6,81^ Functional disability is also higher in those with hallucinations than those without hallucinations in the context of psychiatric disorders such as dissociative identity disorder,^44^ PTSD,^75^ anorexia nervosa,^78^ and narcolepsy. In such cases, hallucinations may point to a more severe form of the disorder. Alternatively, hallucinations may be an experience that restricts patients’ recovery while not being intrinsic to that particular disorder, just as other factors such as educational level and social support will influence prognosis. Altogether, the value of hallucinations may be greater as an indicator of vulnerability or risk than as a diagnostic marker.

## Limitations and future directions

Several limitations apply to the present study. Our inclusion criteria limit the parameters and features investigated to those that were reported in these 43 articles. The absence of a feature in table 1 does not entail that it is a not a feature of the disorder, but rather indicates a lack of evidence on which to make comparisons. The range of features reviewed here is also necessarily limited. First, features tend to cluster in similar dimensions (physical properties, control, frequency, complexity, emotional reaction, special significance, etc.), which may exaggerate similarities across groups of interest. Second, some features, such as the presence of multiple personified voices^96^, frequency, or the contents of visual hallucinations,^81^ are not represented. Third, these features might not be the best way of describing psychopathological phenomena as natural kinds, but may rather represent categories imposed by psychiatric convention.^98^ Our analysis focused on comparison studies involving 2 or more groups, which have generally adopted traditional approaches to symptom assessment. Other approaches that have used qualitative interviews and phenomenological approaches have revealed additional variables that do not figure in the above contrasts. It is possible that studies targeting variables such as changed aspects of self-consciousness, disturbed sense of internal space,^99^ bodily sensations,^96^ and thought-like properties of voices,^96^ may reveal hallucination features that are more discriminative of psychosis than those considered here. Furthermore, the present analysis might have had a different outcome if we had seeded our comparisons with a diagnostic group other than schizophrenia, such as Parkinson’s disease, where the features for comparison might have been significantly different. We could have also have adopted different criteria, such as comparison of hallucinations among 2 or more diagnostic groups without the requirement that one be schizophrenia. A final limitation is that the studies reviewed adopted widely varying measures and designs. Further progress in transdiagnostic comparison of hallucination features arguably requires consistency of measures across the groups studied.^100^

Finally, we consider implications for cognitive and neuroscientific models of hallucinations. We noted at the outset that there is very limited evidence in the cognitive and neuroscientific domains on which to base transdiagnostic comparisons of hallucinations. A further issue concerns the problem of mapping cognitive profiles of different groups to specific features of hallucinations, as opposed to their mere presence or absence. Mostly such research compares diagnostic groups with healthy controls or (increasingly) uses hallucination incidence as an independent variable within a particular diagnostic group: eg, comparing schizophrenia patients with and without hallucinations.

For these and other reasons, a discussion and comparison of cognitive and neurobiological models across diagnostic groups is beyond the scope of the present article. There are well-developed models of hallucinations in schizophrenia^9,11,101,102^ and in neurodegenerative disease such as Parkinson’s disease.^103–105^ A complication is that the models for these disorder groups are rather divergent, with some schizophrenia models proposing hyperactivity of the auditory cortex mediated by dopamine release and Parkinson’s models proposing underactivity of the occipital cortex mediated by acetylcholine. No such models have been developed for other conditions except for nonclinical hallucinations, which some continuum approaches propose can be best understood as variants of schizophrenia models. Given the many featural similarities of hallucinations among the disorders discussed here, further effort is needed in developing and integrating neurocognitive models across diagnostic groups.

## Conclusions

Hallucinations are a feature of human experience that crosses diagnostic category boundaries and straddles the divide between psychopathological and nonclinical experience. They occur widely across diagnostic disorders and their presence or absence is only clinically useful when considered in conjunction with other symptoms and clinically relevant data. There are no “points of rarity” when we address the features of hallucinations in schizophrenia, with the possible exception of age of onset. We recommend that future studies systematically ask about onset, and the conditions and context in which hallucination first occurred.^106,107^ Other features may be particularly valuable in distinguishing other disorders, but more research is needed which pays attention to phenomenological and clinical features of hallucinations and compares them in methodologically robust designs across diagnostic groups and into the nonclinical population.

## Supplementary Material

Supplementary material is available at http://schizophreniabulletin.oxfordjournals.org.

## Funding

Wellcome Trust (WT108720 to C.F.).

## Supplementary Material

Supplementary Data
