# Supplementary material for: Hallucinations: A Systematic Review of Points of Similarity and Difference Across Diagnostic Classes
Source: Schizophr Bull. 2016 Nov 21;43(1):32–43. doi: 10.1093/schbul/sbw132 (PMC5216859; doi:10.1093/schbul/sbw132)
Supplement: Supplementary Data [file supp_43_1_32__index.html]

Hallucinations: A Systematic Review of Points of Similarity and Difference Across Diagnostic Classes — Hallucinations: A Systematic Review of Points of Similarity and Difference Across Diagnostic Classes — Supplementary Data 

# Hallucinations: A Systematic Review of Points of Similarity and Difference Across Diagnostic Classes

## Supplementary Data

Data files

- Supplementary Data - Supplementary Data
