# Supplementary material for: Hallucinations: A Systematic Review of Points of Similarity and Difference Across Diagnostic Classes
Source: Schizophr Bull. 2016 Nov 21;43(1):32–43. doi: 10.1093/schbul/sbw132 (PMC5216859; doi:10.1093/schbul/sbw132)
Supplement: Supplementary Data [file supp_sbw132_Supplementary_Material_Table_1_2.doc]

**Supplementary Material Table 1. Occurrence of hallucinations**

| **Hallucinations in the general (non-clinical) population** | |
| --- | --- |
| Non-clinical causes | General population on a continuum; sleep-related experiences (lucid dreaming, hypnagogic and hypnagogic hallucinations, sleep deprivation);  Induced by hypnosis, trance states; religious and ritual activities; sociocultural expectations and influences; bereavement; sensory isolation and solitary confinement; food deprivation; psychosocial adversity including childhood abuse; fatigue; life-threatening stress. |
| **Hallucinations caused by the effects of substances** | |
| Substance-induced conditions (toxins, drugs and alcohol) | *Intoxication with* stimulants (e.g., amphetamine, cocaine), hallucinogenic drugs (psilocybin, LSD; tryptamine; MDMA), mescaline, inhalants; phencyclidine (PCP), ketamine, cannabis; *Withdrawal* from alcohol, sedatives, hypnotic or anxiolytic medications; *Substance dependence* from alcohol and other drugs; *Side-effect or abuse* of glucocorticosteroids, attention-deficit/hyperactivity disorder (ADHD), antimalarials, antiparkinsonian drugs, benzodiazepine, anticonvulsants, antihypertensives, antiarrhythmic agents; antibiotics; anticholinergic agents; antipsychotics; opioids; *Poisoning* from mercury, arsenic, or lead. |
| **Hallucinations in medical and neurological conditions** | |
| Ear and eye disorders | Age-related macular degeneration, cataract, glaucoma or diabetic retinopathy (Charles Bonnet syndrome); tinnitus, hearing impairments or loss, disorder from damage to the auditory pathway or circuitry including brain stem. |
| Metabolic and endocrine disorders | Niemann-Pick type C; Tay Sachs disease; metachromatic leukodystrophy; Wilson’s disease; porphyria; adrenal disorders; hypothyroidism; hyperthyroidism; hypoparathyroidism, hyperparathyroidism; hypocalcaemia; thyrotoxicosis; premenstrual dysphoric disorder. |
| Dietary deficiencies | Vitamin B12 deficiency; vitamin D deficiency; folic acid deficiencies; copper and zinc deficiencies. |
| Congenital, chromosomal and cytogenetic disorders | Velocardiofacial *(*22q11*.2* deletion syndrome); Prader–Willi syndrome; Huntington’s disease; Wilson's disease; Fahr’s disease. |
| Infectious diseases and autoimmune disorders | Neurosyphilis; HIV/AIDS, toxoplasmosis; sarcoidosis, systemic lupus erythematosus; Hashimoto disease; NMDA receptor encephalitis. |
| Neurological lesions, seizures, and cerebrovascular events | Epilepsy; complex seizures; stroke; brain tumors; temporal lobectomy; other lesions involving the occipital and temporo-parietal pathways; mild neurocognitive disorder; vascular hematomas; intracerebral and arteriovenous malformations; meningiomas; encephalitis; traumatic brain injury; concussion syndrome; migraines; delirium. |
| Neurodegenerative disorders | Parkinson’s disease; Alzheimer’s disease; Dementia with Lewy Bodies (DLB); vascular dementia; frontotemporal dementia (FTD); prion disease; progressive supranuclear palsy; multiple sclerosis; pontine degeneration; delirium tremens. |
| **Hallucinations in psychiatric disorders** | |
| Schizophrenia spectrum disorders | Schizophrenia; schizoaffective disorder; delusional disorder; schizoid, paranoid and schizotypal (personality) disorder; schizophreniform disorder; brief psychotic disorder; other specified schizophrenia spectrum and other psychotic disorder.. |
| Affective disorders | Major depression with psychotic features; bipolar disorder type I and II. |
| Personality disorders | Borderline personality disorder |
| Anxiety disorders, stressor-related disorders; dissociative disorders | Anxiety disorders with psychotic features; phobias; dissociative disorders; obsessive-compulsive disorder; Tourette syndrome; post-traumatic stress disorder (PTSD), acute stress disorder; dissociative identity disorder. |
| Neurodevelopmental disorder | Autism; attention-deficit/hyperactivity disorder (ADHD); other disruptive behaviour disorders; other psychopathology including early onset schizophrenia and affective disorder. |
| Eating disorders | Anorexia and bulimia nervosa; narcolepsy; sleep deprivation. |
| Sleep-wake disorders | Narcolepsy; sleep deprivation. |

**Supplementary material 2: List of studies included in systematic review**

| **Authors** | **N SCZ^1^** | **Comparison sample** | **N** |
| --- | --- | --- | --- |
| Aggernaes et al 1972 | 41 | LSD intoxication | 26 |
| Alpert & Silvers 1970 | 45 | Alcohol dependence disorder | 18 |
| Baethge et al 2005 | 130 | Bipolar disorder  Major depression with psychotic features | 65 |
| Bell 1965 | 7 | Cocaine dependence disorder | 7 |
| Bliss 1962 | * | LSD intoxication | * |
| Bowman 1931 | 1408 | Bipolar disorder | 1009 |
| Brett et al 2015 | 37 | Ultra-high risk (UHR) for psychosis | 21 |
| Chaturvedi & Sinha 1990 | 18 | Affective disorders mixed | 30 |
| Cottam et al 2011 | 14 | Non-clinical (religious Christians) | 15 |
| Cutting 1987 | 74 | Medical and neurological conditions mixed | 74 |
| Daalman et al 2011 | 111 | Non-clinical | 118 |
| Davies et al 2011 | 18 | Non-clinical (evangelical Christians) | 29 |
| Dorahy et al 2009 | 34 | Dissociative identity disorder | 29 |
| Eagles et al 1990 | 88 | Anorexia nervosa | 73 |
| Frieske & Wilson 1966 | 50 | Medical and neurological conditions mixed Affective disorder | 30  15 |
| Fortuyn et al 2009 | 102 | Narcolepsy | 60 |
| Goodwin et al 1971 | 45 | Affective disorders  Alcohol dependence disorder  Medical and neurological conditions mixed | 28  27  9 |
| Hamner et al 2000 | 40 | Post-traumatic stress disorder (PTSD) | 40 |
| Hepworth et al 2013 | 23 | Borderline personality disorder | 10 |
| Honig et al 1998 | 18 | Non-clinical  Dissociative identity disorder | 15  15 |
| Jessop et al 2008 [^61^](#_ENREF_61) | 5 | Post-traumatic stress disorder (PTSD) | 13 |
| Johns et al 2002 | 14 | Neurological (tinnitus) | 16 |
| Johnstone et al 1988 | 43 | Neurological disorders mixed  Affective disorders | 22  44 |
| Junginger & Frame 1985 | 52 | Affective disorders | 41 |
| Kimhy et al 2007 | 34 | Ultra high risk (UHR) of psychosis | 32 |
| Kingdon et al 2010 | 59 | Borderline personality disorder | 33 |
| Lindal et al 1994 | 19 | Non clinical | 187 |
| Leudar et al 1997 | 14 | Non clinical | 13 |
| Lowe 1973 | 15 | Bipolar disorder  Neurological disorders mixed | 15 |
| Malitz et al 1962 | 100 | LSD intoxication | 34 |
| Mitchell & Vierkant 1991 | 100 | Cocaine withdrawal | 100 |
| Mott et al 1965 | 50 | Alcohol dependence disorder | 50 |
| Núñez & Gurpegui 2002 | 35 | Cannabis abuse | 26 |
| Okulate & Jones 2003 | 76 | Affective disorders mixed | 13 |
| Perez et al, 1985 | 7 | Epilepsy | 17 |
| Peters et al 2016 | 84 | Non-clinical | 92 |
| Shinn et al 2012 | 325 | Bipolar disorder | 244 |
| Slater & Beard 1963 | 46 | Epilepsy | 15 |
| Slotema et al 2012 | 51 | Borderline personality disorder | 38 |
| Standage 1973 | 2 | Epilepsy | 6 |
| Tschoeke et al 2014 | 21 | Borderline personality disorder | 23 |
| Waters et al 2014 | α | Parkinson’s disease  Dementia Lewy Body | α |
| Young 1972 | 20 | LSD intoxication | 20 |

**^1^** Schizophrenia-spectrum (primarily schizophrenia, schizoaffective); ^*^ sample size not reported; ^α^ Comparisons made from literature review.
